# Supplementary material for: Complex‐centric proteome profiling by SEC‐SWATH‐MS
Source: Mol Syst Biol. 2019 Jan 14;15(1):e8438. doi: 10.15252/msb.20188438 (PMC6346213; doi:10.15252/msb.20188438)
Supplement: Supplementary file 8 — Dataset EV7 [file MSB-15-e8438-s008.zip › feature_plots_string/Q9Y5K5.pdf]

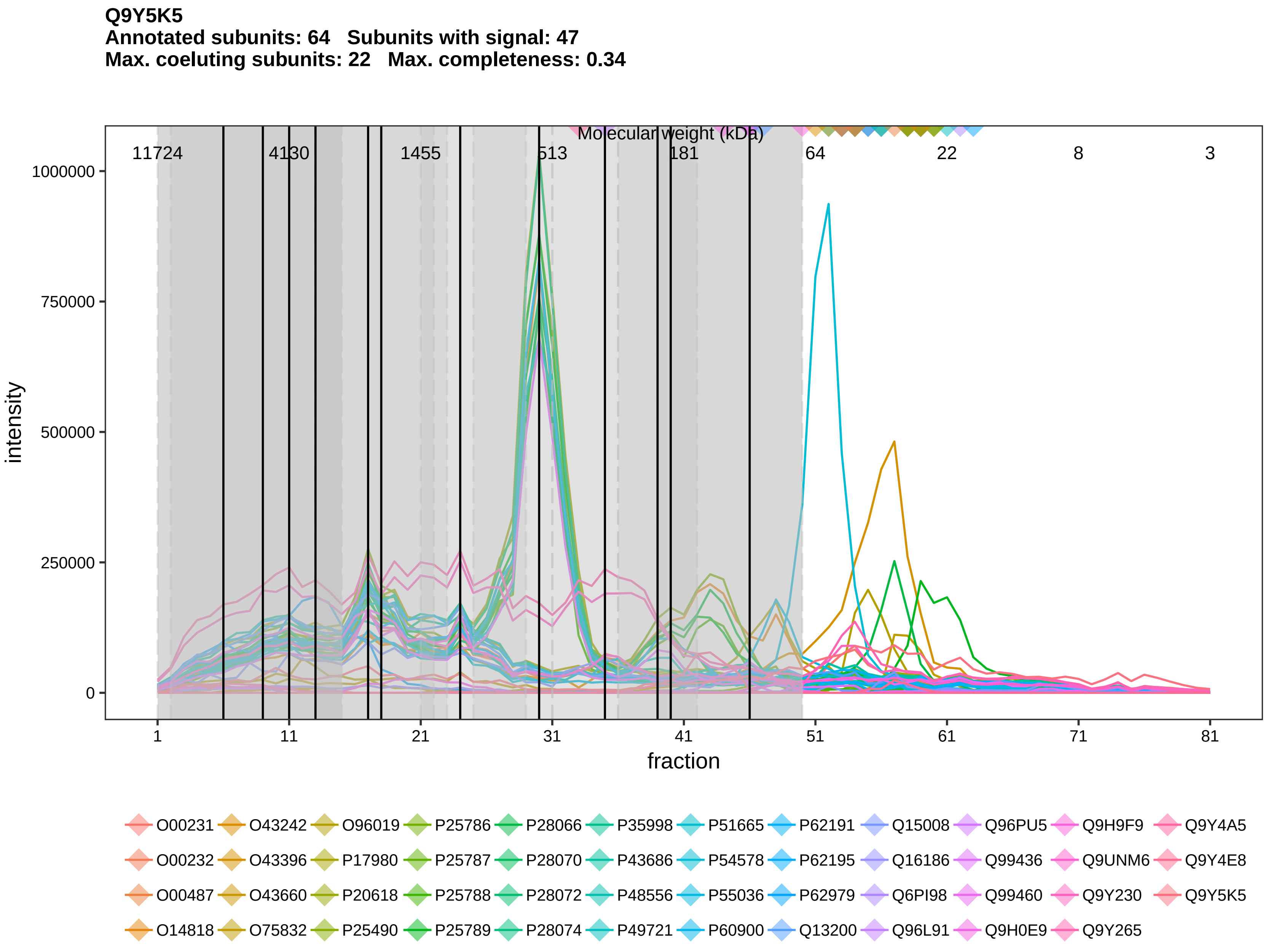

Q9Y5K5
Annotated subunits: 64 Subunits with signal: 47
Max. coeluting subunits: 22 Max. completeness: 0.34
Molecular weight (kDa)
11724
4130
1455
513
181
64
22
8
3
intensity
1000000
750000
500000
250000
0
fraction
1
11
21
31
41
51
61
71
81
O00231
O43242
O96019
P25786
P28066
P35998
P51665
P62191
Q15008
Q96PU5
Q9H9F9
Q9Y4A5
O00232
O43396
P17980
P25787
P28070
P43686
P54578
P62195
Q16186
Q99436
Q9UNM6
Q9Y4E8
O00487
O43660
P20618
P25788
P28072
P48556
P55036
P62979
Q6PI98
Q99460
Q9Y230
Q9Y5K5
O14818
O75832
P25490
P25789
P28074
P49721
P60900
Q13200
Q96L91
Q9H0E9
Q9Y265
